# Twenty-four-hour Skin Temperature Rhythms in Young People With Emerging Mood Disorders: Relationships With Illness Subtypes and Clinical Stage

**DOI:** 10.1177/07487304251328501

**Published:** 2025-04-26

**Authors:** Mirim Shin, Joanne S. Carpenter, Shin H. Park, Connie Janiszewski, Emiliana Tonini, Sarah McKenna, Gabrielle Hindmarsh, Frank Iorfino, Alissa Nichles, Natalia Zmicerevska, Elizabeth M. Scott, Benjamin L. Smarr, Ian B. Hickie, Jacob J. Crouse

**Affiliations:** * Youth Mental Health and Technology, Brain and Mind Centre, The University of Sydney, Sydney, NSW, Australia; † Shu Chien-Gene Lay Department of Bioengineering, University of California San Diego, San Diego, California, USA; ‡ Halıcıoğlu Data Science Institute, University of California San Diego, San Diego, California, USA

**Keywords:** bipolar, chronobiology, rhythms, mental, psychiatric, depression, actigraphy

## Abstract

While circadian disruptions are common in some sub-groups of youth with mood disorders, skin temperature rhythms in these cohorts are understudied. We examined 24-h wrist skin temperature rhythms in youth with emerging mood disorders, exploring associations with clinical stage and proposed illness subtypes. Youth (*n* = 306, 23.42 ± 4.91 years, 65% females) accessing mental health care and 48 healthy controls (23.44 ± 3.38 years, 60% females) were examined. Skin temperature parameters including rhythm-adjusted mean temperature, inter-daily stability (day-to-day consistency), intra-daily variability (rhythm fragmentation), and peak temperature time were derived from a wearable sensor. Based on our illness trajectory-pathophysiology model, participants were classified by mood disorder subtypes (“hyperarousal-anxious” [*n* = 209], “neurodevelopmental-psychosis” [*n* = 40], or “circadian-bipolar spectrum” [*n* = 43]), as well as by clinical stage (subthreshold disorders classed as 1a or 1b [*n* = 47, 173, respectively], and full-threshold disorders as 2+ [*n* = 76]). Compared to controls, youth with mood disorders had delayed, less stable, and more variable skin temperature rhythms, indicated by lower rhythm-adjusted mean skin temperature (29.94 ± 0.10 °C vs 31.04 ± 0.25 °C, *p* < 0.001), delayed peak timing (0533 ± 0014 vs 0332 ± 0036, *p* = 0.002), reduced inter-daily stability (*p* = 0.009), and increased intra-daily variability (*p* = 0.020). Peak skin temperature also occurred later relative to sleep midpoint (0.31 ± 0.14 vs −0.48 ± 0.35 radians, *p* = 0.037). The “circadian-bipolar spectrum” subtype exhibited lower relative amplitude (0.07 ± 0.005 vs 0.08 ± 0.002 [hyperarousal-anxious] and 0.09 ± 0.005 [neurodevelopmental-psychosis], *p* = 0.039), with no delay in sleep midpoint. Clinical stages were not associated with differences in skin temperature parameters. These findings highlight the potential of use of 24-h skin temperature rhythms as a non-invasive biomarker of circadian disturbances in youth with emerging mood disorders. The observed disruptions in temperature patterns and rhythmicity support the notion that disrupted circadian rhythms may mediate the onset or illness course of some subgroups of youth with emerging major mood disorders.

The profound global burden of disease caused by depressive and bipolar disorders (GBD 2019 Mental Disorders Collaborators, 2020; GBD 2019 Mental Disorders Collaborators, 2022; Kieling et al., 2024) makes the search for causal mechanisms of these disorders an urgent priority (Hickie et al., 2013b; Hickie et al., 2019; Crouse et al., 2021; McGorry et al., 2024). One potential pathophysiological mechanism is circadian disruption. Circadian rhythms are endogenous, self-sustained ~24-h cycles that synchronize behavior and physiological processes in response to the Earth’s day/night cycle (Hut and Beersma, 2011). Circadian rhythm disruptions have been consistently linked with depressive and bipolar disorders across many studies globally (Carpenter et al., 2017; Robillard et al., 2018; McCarthy et al., 2022; Scott et al., 2022). These 24-h biological rhythms are regulated by various factors, including light exposure, feeding, and hormonal signals such as melatonin secretion (Carpenter et al., 2021). Body temperature is another well-recognized marker and output associated with circadian rhythms. Compared to healthy controls, people with depressive and bipolar disorders often show altered core body temperature patterns, including blunted, phase-shifted, or irregular temperature curves (Elsenga and Van den Hoofdakker, 1988; Szuba et al., 1997; Avery et al., 1999; Hasler et al., 2010), which appear primarily due to higher nocturnal temperatures (von Zerssen et al., 1985; Souêtre et al., 1989; Tsujimoto et al., 1990; Daimon et al., 1992). However, the literature is somewhat mixed on whether there are differences in the phase of core body temperature in people with mood disorders compared to healthy controls.

Nonetheless, these temperature alterations may be clinically important. For instance, a larger time-lag (or “phase angle difference”) between midsleep and core body temperature minimum has been reported to be associated with greater depressive symptoms in people with “nonseasonal” depressive disorders (Hasler et al., 2010). And notably, elevated nocturnal temperatures have been associated with greater symptom severity, with temperature amplitudes shown in some studies to increase upon clinical recovery (Avery et al., 1982; Souetre et al., 1988; Szuba et al., 1997). However, measurement of core body temperature is typically invasive and confined to tightly controlled laboratory settings. This has limited the ecological validity of such findings and the exploration of core body temperature in clinical research (Lorenz et al., 2019).

To overcome these limitations, some studies have explored the use of skin temperature as a non-invasive alternative, which can be measured continuously via wearables in real-world conditions. Peripheral skin temperature, the daily waveform of which is phase-advanced and inversely related to core body temperature, plays a key role in thermoregulation via heat loss from the extremities (Krauchi and Wirz-Justice, 1994; Sarabia et al., 2008). A recent report from the *TemPredict Study* found that people with depressive disorders exhibit reduced asleep-awake temperature differences (including elevated distal body temperature during sleep); the study also reported that elevated body temperature while awake (i.e., reduced daily rhythm amplitude) was nominally associated with greater depressive symptom severity (Mason et al., 2024). Despite these findings, research on 24-h (circadian) skin temperature rhythms in people with mood disorders remains limited and inconsistent. Although some studies report differences in skin temperature rhythm amplitude between people with depressive disorders and healthy controls (Moraes et al., 2013)—particularly when excluding those taking antidepressants (Lorenz et al., 2019)—other studies (especially in adolescents) have not observed such associations (Kuula et al., 2022). These inconsistencies highlight the need for further investigation into peripheral skin temperature rhythms in depressive and bipolar disorders, particularly as a non-invasive marker of circadian function.

Therefore, this study aims to compare 24-h skin temperature rhythms:

1. Between youth with emerging mood disorders and healthy controls. We hypothesize that the mood disorder group will exhibit reduced temperature amplitude and delayed temperature rhythms compared to healthy controls.
2. Between 3 illness subtypes, whereby cases are assigned to a clinical phenotype associated with a proposed underlying pathophysiology and illness trajectory (Hickie et al., 2013a; Carpenter et al., 2019): (a) “hyperarousal-anxious”; (b) “neurodevelopmental-psychosis”; and (c) “circadian-bipolar spectrum.” We hypothesize that the “circadian-bipolar spectrum” subtype will exhibit reduced temperature amplitude and delayed temperature rhythms compared to the other 2 subtypes.
3. Between clinical stages according to the transdiagnostic clinical staging model (Shah et al., 2020; Scott et al., 2024). We hypothesize that later clinical stages will have more pronounced reductions in temperature amplitude and delays in temperature rhythms.

## Materials And Methods

### Participants

This study included young people (aged 13-35 years) seeking mental health care at primary care–based early intervention services (*headspace*) and healthy controls from the same local community without any personal history of a mental disorder. Recruitment occurred between October 2008 and January 2024 across 2 transdiagnostic studies at the University of Sydney’s Brain and Mind Centre: *Youth Mental Health Follow-Up Study (YMH)* (Lee et al., 2018) and *Neurobiology Youth Follow-Up Study* (Nichles et al., 2021). Detailed information about these studies is available in previous publications (Lee et al., 2018; Carpenter et al., 2020; Nichles et al., 2021). Briefly, these studies involved assessments across clinical, self-report, neurocognitive, sleep-wake (e.g., wearable sensor data), and biological domains (e.g., blood sampling) at one or multiple time points. Participants were excluded if they had insufficient English proficiency, an intellectual disability, and in addition, for the YMH study, a history of neurological disease, medical conditions affecting brain function, recent electroconvulsive therapy, or current substance dependence.

Participants included in the current analysis had at least 5 valid days of wearable sensor data (skin temperature and motor activity recording) from a GENEActiv device, as well as clinical assessments conducted within 6 months of the wearable sensor recording. Healthy controls, recruited from the same metropolitan area and without a personal history of a mental disorder, also completed the wearable sensor component. Ethics approval was obtained from the University of Sydney Human Research Ethics Committee (2012/1631) for the YMH study and the Sydney Local Health District Human Research Ethics Committee (2020/ETH01272) for the Neurobiology study. All participants provided written informed consent; for those under 16 years, parental/guardian consent was also obtained.

### Measures

Trained clinical researchers administered a series of multimodal assessments, including the following measures reported in this paper:

- Demographics: Age, sex, and body mass index (BMI; direct or self-reported weight (kg) ÷ height (m)^2^). Notably, 30% of BMI data are missing (*n* = 105).
- Wearable Sensor Data (see below for details): Participants wore a wrist-worn sensor device (GENEActiv, Activinsights, Kimbolton, UK) for 5-24 days (median 13 days). Participants were instructed to wear the device on their non-dominant wrist and remove it only for showering, bathing, or swimming. Raw acceleration data were collected at either 30 or 50 Hz (difference due to protocol updates), while temperature was measured every 30 s with a resolution of 0.25 °C and a range of 0-60 °C using a manufacturer-calibrated digital temperature sensor. Where multiple wearable recordings were available for one participant, the earliest recording was included.

The following assessments were conducted only for people with emerging mood disorders:

- Mental health assessments: Brief Psychiatric Rating Scale (BPRS) (Overall and Gorham, 1962), Young Mania Rating Scale (YMRS) (Young et al., 1978), and the Social and Occupational Functioning Assessment Scale (SOFAS) (Goldman et al., 1992);
- Illness subtypes: Our team has proposed 3 illness subtypes, based on adolescent-onset clinical phenotypes linked to potential pathophysiological mechanisms and illness trajectories (Hickie et al., 2013a; Carpenter et al., 2019; Hickie et al., 2019). Youth with significant manic-like symptoms or atypical features (e.g., reduced activation, low energy, prolonged fatigue) were allocated to the “circadian–bipolar spectrum” subtype. Youth with a primary psychotic disorder or a history of childhood-onset developmental difficulties (e.g., autism spectrum disorder, specific learning disability) were allocated to the “neurodevelopmental-psychosis” subtype. Youth reporting anxiety symptoms and depressive disorder symptoms, or without clear evidence consistent with one of the other subtypes, were allocated to the “hyperarousal-anxious depression” subtypes.
- Clinical stage: Clinical stage was based on the transdiagnostic model, encompassing stages 1a (“seeking help” for those in the earliest phases with non-specific clinical presentation); 1b (“attenuated syndrome” for those at greater risk with subthreshold clinical presentations); and 2+ (for those who have reached a threshold for progressive or recurrent disorders) (Hickie et al., 2013a; Iorfino et al., 2019; Shah et al., 2020; Scott et al., 2024).

### Wearable Sensor Data and Processing

Raw GENEactiv data was processed using an open-source R package, GGIR (version 3.0.5), developed for multi-day accelerometer data processing (Migueles et al., 2019). Post-processing was completed using the package mMARCH.AC (version 2.9.2) according to protocols established for the “Mobile Motor Activity Research Consortium for Health” (mMARCH) (Guo et al., 2023).

#### Skin Temperature Data Processing

Temperature data were collected at 30-s intervals and processed independently of acceleration data. During GGIR processing, these raw measurements were aggregated into 15-min intervals using arithmetic means. We observed that when participants resumed wearing the device after removal, temperature readings were unreliably low for a period of time. Therefore, to maintain data quality, entire days were excluded from analysis if any non-wear periods were detected by GGIR. For inclusion in the primary analysis, participants were required to have at least 2 days of complete skin temperature data (Thomas and Burr, 2008). A secondary analysis including only participants with 5 or more days of data is presented in the Supplementary Materials. The reported parameters included the following:

- Cosinor-based parameters: Although skin temperature and other circadian rhythms are not perfect sine waves, we employed cosinor analysis following established chronobiological methods (Cornelissen, 2014) to generate parameters comparable with existing literature on skin temperature rhythms (Sarabia et al., 2008).
- Midline Estimating Statistics of Rhythm (Mesor): A circadian rhythm-adjusted mean temperature, representing the central value around which temperature oscillates. Mesor is based on the parameters of a cosine function fitted to the raw data. For rhythmic processes, the mesor often provides a more appropriate unbiased estimate of central tendency than does the arithmetic mean of the raw data.
- Amplitude: A measure of half the range of predictable temperature variation within a cycle.
- Non-parametric parameters (Van Someren et al., 1999; Tai et al., 2023):
- Relative Amplitude (RA): $RA=\frac{\left(M5-L10\right)}{\left(M5+L10\right)},$ where M5 and L10 are the highest 5-h moving mean and the lowest 10-h moving mean of skin temperature. These moving means were derived minutewise across days to form an average 24-h pattern across the recorded periods. Higher values indicate stronger rhythmicity.
- Acrophase: A measure of the time of the peak values recurring in each cycle. This parameter shows a unit of radian which represents time to reach the peak. Acrotime, which represents acrophase in hours, is also provided.
- Inter-daily Stability (IS): a non-parametric measure of the consistency of the temperature rhythm across days. Higher values indicate greater consistency of the rhythm.
- Intra-daily Variability (IV): a non-parametric measure indicating the fragmentation of the temperature rhythm. Higher values indicate greater fragmentation of the rhythm.

The 24-h cosinor analysis was performed in 2 key steps. First, using the “ActCosinor” function, we averaged the 15-min temperature measurements across days for each participant and fitted a cosine curve with a 24-h period. Then, using the “GLMMcosinor” R package, we fitted a mixed-effects cosinor model that accounts for group differences (Control vs Mood disorder cases, Tripartite groups, and Clinical Stages). The model uses a fundamental cosine function:

Y(t)=M+A*cos(2π*t/24+φ)

where:

M = mesor

A = amplitude

φ = acrophase

t = time in hours (measured in 15-min intervals over 24 h)

The model was fitted using the cglmm function with a fixed 24-h period and group-specific parameters, allowing for comparison of circadian characteristics between different clinical groups while accounting for the cyclic nature of the temperature rhythm.

#### Validation of GENEActiv Temperature Measurement

To validate the GENEActiv sensor for skin temperature measurement, we conducted a comparison study with iButton sensors (DS1922L, Maxim Integrated Products, San Jose, CA), which are widely validated peripheral temperature sensors in sleep and circadian research (van Marken Lichtenbelt et al., 2006; Hasselberg et al., 2013; Shin et al., 2017). A subset of participants (*n* = 37) wore both devices simultaneously with the iButton placed on the inner forearm (closest to the wrist) and GENEActiv on the wrist, under controlled conditions (<30 lux) for approximately 10 h (from 8 h before to 2 h after their habitual sleep time). Bland-Altman analysis and time series comparisons of the simultaneous recordings were conducted to assess agreement between devices (see Supplementary Materials Figures S1-S3).

##### 24-h sleep-wake parameters

These parameters are estimated from activity and are explored in a larger sample focused on sleep-wake cycles in a separate publication. GGIR and mMARCH.AC algorithms were used to generate sleep (van Hees et al., 2018), physical activity (van Hees et al., 2013), and circadian parameters (Guo et al., 2023). The following parameters are reported here primarily for comparison with skin temperature:

- Sleep efficiency: percentage of time estimated as asleep between sleep onset and wake time.
- Sleep midpoint: halfway point between sleep onset and wake time.
- Sleep duration: number of minutes estimated as asleep per 24-h period.
- Sleep Regularity Index (SRI): a metric comparing the similarity of sleep patterns from one day to the next, calculated using the R package sleepreg (version 1.3.5) (Windred et al., 2024) based on sleep-wake estimates by epoch from GENEActiv GGIR output. A minimum of 5 overlapping days was required to calculate the SRI score.
- M10 time: time of onset of the most active 10 h.
- Moderate/vigorous physical activity: sum of 1-min epochs in which gross motor activity was larger than 100 mg.
- Total activity count: total gross motor activity count per day.
- Phase angle: time difference between sleep midpoint and acrotime of skin temperature was calculated by subtracting the time of sleep midpoint from skin temperature acrotime.

### Statistical Analysis

Statistical analyses were conducted using R (4.2.3) in RStudio (2024.04.2 + 764) and SPSS (version 27.0). Normality of wearable-derived parameter distributions was assessed using QQ plots and Shapiro-Wilk tests. Demographic and clinical variable comparisons between youth with mood disorders and healthy controls were conducted using the Mann–Whitney *U* test or χ^2^ test. The Kruskal-Wallis rank sum test or χ^2^ test was used to examine differences among illness subtypes and clinical stages. Analysis of covariance (ANCOVA) was employed to compare each wearable-derived metric (skin temperature and sleep-wake parameters) between youth with mood disorders and healthy controls, as well as across illness subtypes and clinical stages (adjusting for age and sex). Non-normally distributed variables were Box-Cox transformed prior to ANCOVA. For significant results from the Kruskal-Wallis tests, Dunn’s post hoc tests were performed. Bonferroni post hoc pairwise comparisons were conducted for ANCOVA results. A *p*-value < 0.05 was considered significant. For Mann–Whitney *U* test and Krskal-Wallis test results, data are presented as mean ± standard deviation, whereas data are presented as estimated mean ± standard errors for ANCOVA results. As supplementary analyses, correlations between skin temperature, sleep/wake parameters, and mental health symptoms were explored. As a supplementary analysis, an additional ANCOVA was conducted, controlling for age, sex, and BMI (see Tables S3-S5).

## Results

### Skin Temperature Rhythms in Youth With Mood Disorders Versus Healthy Controls

Among the young people who have minimum 2 days of completed 24-h skin temperature data included in this study (67% aged 12-25 years; 95% aged 12-30 years), 306 were people with emerging mood disorders (23.42 ± 4.91 years, 65% females) and 48 were healthy controls (24.44 ± 3.38 years, 60% females). The mood disorder group had a significantly higher BMI than the control group (24.70 ± 5.18 kg/m^2^ vs 22.15 ± 3.62 kg/m^2^, *p* = 0.047).

**Figure 1.**
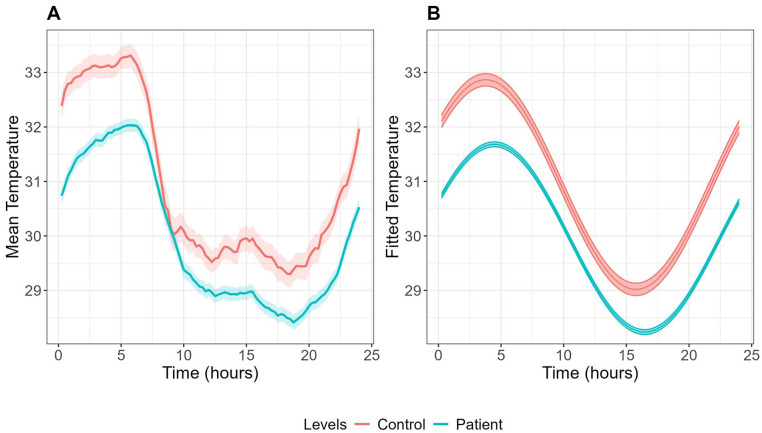
Mean (A) and fitted cosinor curves (B) for wrist skin temperature over 24 h in youth with mood disorders (*n* = 306) and healthy controls (*n* = 48). Temperature data were averaged across all available wear days for each participant to create these curves.

As shown in Table 1 and Figure 1, several significant differences in skin temperature parameters were found between groups after controlling for sex and age. Youth with mood disorders exhibited a lower temperature mesor (29.94 ± 0.10 °C vs 31.04 ± 0.25 °C, *p* < 0.001) and a significantly delayed acrophase (−1.45 ± 0.06 vs −0.93 ± 0.16 radian, corresponding to 0533 ± 0014 vs 0332 ± 0036, *p* = 0.002), compared to healthy controls. The mood disorder group also showed lower inter-daily stability (IS; 0.52 ± 0.01 vs 0.60 ± 0.03, *p* = 0.009) but higher intra-daily variability (IV; 1.51 ± 0.02 vs 1.37 ± 0.06, *p* = 0.020), relative to healthy controls.

**Table 1.** Demographics, skin temperature, and sleep-wake parameters in youth with mood disorders versus healthy controls.

	Healthy Controls (*n* = 48)	Mood Disorders (*n* = 306)	Mann-Whitney/χ^2^
Age (years)	24.44 (3.38)	23.42 (4.91)	*U* = 6204.5, 0.083
Sex (Female (%))	29F (60)	200F (65)	χ2(1) = 0.444, 0.505
Recording duration (days)	9.21 (2.45)	8.64 (3.57)	*U* = 6470.5, 0.183
**BMI (kg/m** ^**2**^ **)** **(%missing BMI)**	**22.15 (3.62)** **(63%)**	**24.70 (5.18)** **(25%)**	***U*** **=** **1495.5, 0.047**
			ANCOVA
*Skin temperature parameters*			
**Mesor (°C)**	**31.04 (0.25)**	**29.94 (0.10)**	***F*(1,350)** **=** **16.29, < 0.001**
Amplitude (°C)	2.07 (0.14)	1.97 (0.06)	*F*(1,350) = 0.40, 0.526
**Acrophase (radians)**	**-0.93 (0.16)**	**-1.45 (0.06)**	***F*(1,350)** **=** **9.81, 0.002**
**IS (dimensionless, 0-1)**	**0.60 (0.03)**	**0.52 (0.01)**	***F*(1,350)** **=** **7.00, 0.009**
**IV (dimensionless, 0-2)**	**1.37 (0.06)**	**1.51 (0.02)**	***F*(1,350)** **=** **5.45, 0.020**
RA (dimensionless, 0-1)	0.07 (0.004)	0.08 (0.002)	*F*(1,350) = 0.61, 0.437
*Sleep-wake parameters*			
Sleep efficiency (%)	84.8 (0.8)	84.3 (0.3)	*F*(1,350) = 0.38, 0.537
Sleep midpoint (time)	0403 (0013)	0409 (0005)	*F*(1,350) = 0.20, 0.652
Sleep duration (h)	7.22 (0.16)	7.36 (0.06)	*F*(1,350) = 0.72, 0.396
**SRI (0-100)**	**54.85 (2.13)**	**46.84 (0.84)**	***F*(1,350)** **=** **12.24, < 0.001**
M10 time (time)	1512 (0011)	1506 (0004)	*F*(1,350) = 0.25, 0.619
MVPA (minutes/day)	87.05 (5.90)	77.36 (2.33)	*F*(1,350) = 2.33, 0.128
**TAC (counts/day)**	**37,056.88 (1593.95)**	**33,615.76 (629.76)**	***F*(1,350)** **=** **4.03, 0.046**
**Phase angle (radians)**	**-0.48 (0.35)**	**0.31 (0.14)**	***F*(1,350)** **=** **4.39, 0.037**

For Mann–Whitney/χ^2^, data are presented as mean (standard deviation). For ANCOVA results, data are presented as estimated mean (standard error). IS = inter-daily stability; IV = intra-daily variability; RA = relative amplitude (M5/L10); SRI = Sleep Regularity Index; MVPA = moderate/vigorous physical activity; TAC = total activity count.

Regarding sleep-wake parameters, youth with mood disorders exhibited significantly lower SRI (46.84 ± 0.84 vs 54.85 ± 2.13, *p* < 0.001) and lower total activity count (33,615.76 ± 629.76 vs 37,056.88 ± 1593.95 counts per day, *p* = 0.046), compared to controls. Notably, the phase angle was significantly larger in the mood disorder group (0.31 ± 0.14 vs −0.48 ± 0.35 radian, *p* = 0.037), indicating a later peak of skin temperature relative to sleep midpoint, compared to healthy controls.

### Skin Temperature Rhythms Across Illness Subtypes

**Figure 2.**
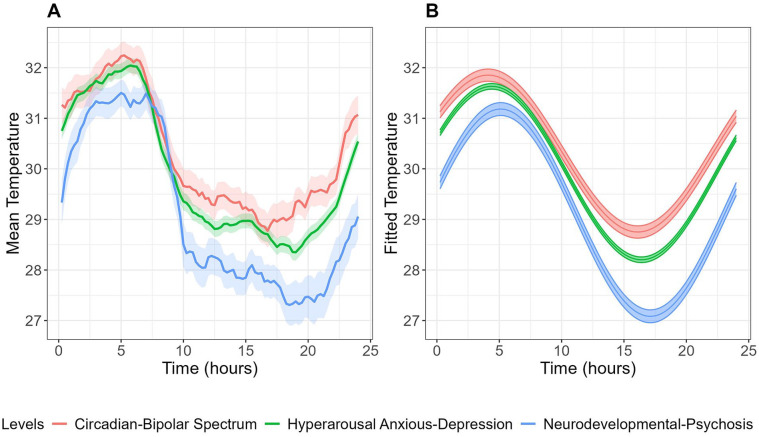
Mean (A) and fitted cosinor curves (B) for circadian wrist skin temperature over 24 h across illness subtypes. Temperature data were averaged across all available wear days for each participant to create these curves.

Table 2 summarizes the skin temperature and sleep-wake characteristics between illness subtypes. Figure 2 displays the mean skin temperature along with fitted cosinor curves for each subtype. A demographic and clinical comparison of these subtypes is provided in Table S1. Among skin temperature parameters, the “circadian-bipolar spectrum” subtype showed significantly higher mesor (30.27 ± 0.27 °C) but lower RA (0.07 ± 0.005; indicating weaker rhythmicity) compared to “neurodevelopmental-psychosis” (29.27 ± 0.28 °C and 0.09 ± 0.005, respectively) (Table 2).

**Table 2.** Skin temperature and sleep-wake parameters across illness subtypes.

	Hyperarousal Anxious-Depression (*n* = 209)	Circadian-Bipolar Spectrum (*n* = 43)	Neurodevelopmental-Psychosis (*n* = 40)	ANCOVA
*Skin temperature parameters*				
**Mesor (°C)**	**29.90 (0.12)**	30.27 (0.27)*	29.27 (0.28)*	***F*(2,287)** **=** **3.38, 0.036**
Amplitude (°C)	1.97 (0.07)	1.84 (0.15)	2.27 (0.16)	*F*(2,287) = 2.03, 0.134
Acrophase (radians)	-1.47 (0.08)	-1.44 (0.18)	-1.40 (0.18)	*F*(2,287) = 0.07, 0.933
IS (0-1)	0.51 (0.01)	0.53 (0.03)	0.56 (0.03)	*F*(2,287) = 1.03, 0.358
IV (0-2)	1.51 (0.03)	1.59 (0.06)	1.41 (0.06)	*F*(2,287) = 1.97, 0.141
**RA (0-1)**	**0.08 (0.002)**	0.07 (0.005)*	0.09 (0.005)*	*F*(2,287) = 3.27, **0.039**
*Sleep-wake parameters*				
Sleep efficiency (%)	84.1 (0.04)	85.0 (0.08)	84.5 (0.09)	*F*(2,287) = 0.52, 0.594
**Sleep midpoint (time)**	0404 (0006)*	**0357 (0014)**	0445 (0014)*	*F*(2,287) = 3.80, **0.023**
Sleep duration (h)	7.26 (0.08)	7.68 (0.18)	7.57 (0.18)	*F*(2,287) = 3.01, 0.051
SRI (0-100)	45.91 (1.06)	47.85 (2.34)	48.58 (2.44)	*F*(2,287) = 0.69, 0.505
M10 time (time)	1501 (0006)	1506 (0012)	1534 (0013)	*F*(2,287) = 2.77, 0.064
MVPA (minutes/day)	78.90 (2.84)	71.18 (6.28)	67.29 (6.54)	*F*(2,287) = 1.71, 0.183
TAC (counts/day)	34,023.33 (770.32)	32,327.36 (1705.29)	30,827.71 (1774.70)	*F*(2,287) = 1.58, 0.209
*Phase angle (radians)*	0.41 (0.18)	-0.11 (0.40)	0.60 (0.41)	*F*(2,287) = 0.92, 0.402

Data are presented as estimated mean (standard error). Asterisk (*) indicates significant differences (*p* < 0.05) in post hoc pairwise comparisons between groups. IS = inter-daily stability; IV = intra-daily variability; RA = relative amplitude (M5/L10); SRI = Sleep Regularity Index; MVPA = moderate/vigorous physical activity; TAC = total activity count.

Analysis of sleep-wake parameters revealed that the “neurodevelopmental-psychosis” subtype exhibited a significantly delayed sleep midpoint compared to the “hyperarousal anxious-depression” subtype (0455 ± 0014 vs 0404 ± 0006, *p* = 0.023).

### Skin Temperature Rhythms Across Clinical Stages

**Figure 3.**
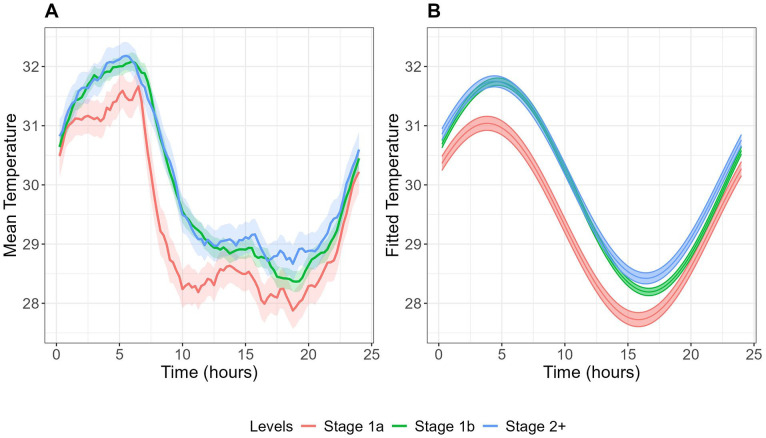
Mean (A) and fitted cosinor curves (B) for circadian wrist skin temperature over 24 h across clinical stages. Temperature data were averaged across all available wear days for each participant to create these curves.

Table 3 summarizes the skin temperature and sleep-wake characteristics of the 3 clinical stages, and Figure 3 shows the corresponding skin temperature patterns. A demographic and clinical comparison of these clinical stages is presented in Table S2.

**Table 3.** Skin temperature and sleep-wake parameters across illness stages.

	Stage 1a (*n* = 47)	Stage 1b (*n* = 173)	Stage 2+ (*n* = 76)	ANCOVA
*Skin temperature parameters*				
Mesor (°C)	29.36 (0.26)	29.95 (0.14)	30.14 (0.21)	*F*(2,291) = 2.81, 0.062
Amplitude (°C)	1.87 (0.15)	2.01 (0.08)	1.99 (0.12)	*F*(2,291) = 0.35, 0.704
Acrophase (radians)	-1.37 (0.17)	-1.45 (0.09)	-1.50 (0.13)	*F*(2,291) = 0.18, 0.834
IS (0-1)	0.50 (0.03)	0.53 (0.02)	0.51 (0.02)	*F*(2,291) = 0.34, 0.715
IV (0-2)	1.55 (0.06)	1.50 (0.03)	1.52 (0.05)	*F*(2,291) = 0.32, 0.724
RA (0-1)	0.08 (0.004)	0.08 (0.002)	0.07 (0.003)	*F*(2,291) = 0.11, 0.898
*Sleep-wake parameters*				
Sleep efficiency (%)	85.9 (0.8)	84.0 (0.4)	84.1 (0.6)	*F*(2,291) = 2.20, 0.112
Sleep midpoint (time)	0353 (0013)	0414 (0007)	0407 (0011)	*F*(2,291) = 0.99, 0.374
**Sleep duration (h)**	6.89 (0.17)*#	7.35 (0.09)*	**7.71 (0.13)#**	***F*(2,291)** **=** **7.39, < 0.001**
**SRI (0-100)**	52.07 (2.22)*#	45.94 (1.15)*	**45.26 (1.74) #**	***F*(2,291)** **=** **3.47, 0.032**
**M10 time (time)**	1429 (0012)*#	1511 (0006)*	**1518 (0009)#**	***F*(2,291)** **=** **6.36, 0.002**
**MVPA** **(minutes/day)**	99.95 (6.09)*#	74.06 (3.16)*	**70.60 (4.78)#**	***F*(2,291)** **=** **8.39, < 0.001**
**TAC (counts/day)**	40,765.35 (1610.02)*#	32,645.42 (834.60)*	**31,257.35 (1262.49)#**	***F*(2,291)** **=** **12.21, < 0.001**
*Phase Angle (radians)*	0.29 (0.38)	0.49 (0.20)	0.03 (0.30)	*F*(2,291) = 0.822, 0.440

Data are presented as estimated mean (standard error). Matching symbols (* or #) indicate significant differences (*p* < 0.05) between groups in post hoc pairwise comparisons. IS = inter-daily stability; IV = intra-daily variability; RA = relative amplitude (M5/L10); SRI = Sleep Regularity Index; MVPA = moderate/vigorous physical activity; TAC = total activity count.

Although no significant differences were found in skin temperature parameters across illness stages, sleep-wake patterns varied significantly. Sleep duration was longer in more advanced illness stages (Stage 1a: 6.89 ± 0.17 h, Stage 1b: 7.35 ± 0.09 h, Stage 2+: 7.71 ± 0.13 h, *p* < 0.001), while SRI showed the opposite pattern, being highest in Stage 1a and lower in more advanced stages (Stage 1a: 52.07 ± 2.22, Stage 1b: 45.94 ± 1.15, Stage 2+: 45.26 ± 1.74, *p* = 0.032). M10 time (peak activity period) showed a progressive delay in more advanced illness stages (Stage 1a: 1429 ± 0012, Stage 1b: 1511 ± 0006, Stage 2+: 1518 ± 0009, *p* = 0.002). Physical activity levels decreased in more advanced illness stages, as evidenced by both moderate/vigorous physical activity (*p* < 0.001) and total activity count (*p* < 0.001) being lower in Stage 2+ and Stage 1b, compared to Stage 1a.

### Goodness of Fit for the Cosinor Analysis

The goodness of fit for the cosinor analysis was assessed using multiple metrics. The Control versus Mood disorder model showed slightly better fit (*R*^2^ = 0.221, root mean square error [RMSE] = 2.316 °C, mean absolute error [MAE] = 1.835 °C) compared to the Tripartite (*R*^2^ = 0.206, RMSE = 2.357 °C, MAE = 1.855 °C) and Clinical Stages models (*R*^2^ = 0.205, RMSE = 2.368 °C, MAE = 1.866 °C). While the *R*^2^ values indicate that approximately 20%-22% of the temperature variance is explained by the models, these results should be interpreted in the context of skin temperature data, which inherently contains substantial variability due to environmental and behavioral factors. The cosinor analysis aims to capture the fundamental 24-h rhythm rather than predict exact temperature values. The relatively consistent fit metrics across all 3 models suggests that the 24-h cosinor function provides a similar level of circadian rhythm characterization regardless of the grouping approach used.

## Discussion

In a large clinical sample of 312 youth with emerging mood disorders and 48 healthy controls, we identified several physiological differences in 24-h (daily) skin temperature rhythmicity. This adds to the growing evidence for circadian pathophysiology as a major mediator of the onset or course of illness in at least some subgroups of youth emerging mood disorders. Furthermore, it encourages the use of wearable-derived skin temperature as a scalable, real-world marker of circadian rhythms.

The hypothesis that wrist skin temperature profiles differ between youth with mood disorders and healthy controls was partially supported, demonstrated by a delayed, less stable, and more variable skin temperature rhythm in the whole mood disorder group. However, no differences in amplitude were observed. As skin temperature differences were not evident across clinical stages, but there was some evidence for their relevance to the “circadian-bipolar spectrum” (as evidenced by the reduced relative amplitude in that sub-group), it would seem that these circadian features are a feature of illness subtype as distinct from course (i.e., recurrence, persistence, or chronicity) of illness.

Overall, youth with mood disorders exhibited lower temperature mesors than healthy controls. Previous research has reported mixed findings regarding mean skin temperature in mood disorder populations. For example, Moraes et al. (2013) observed *lower* nocturnal peripheral temperatures in a small number of older people with depressive disorder (*n* = 20, mean age = 44 years), whereas Mason et al. (2024) reported *higher* distal body temperatures during both wakefulness and sleep in a population-based study of people with self-reported depressive symptoms (*n* > 20,000, mean age = 46.9 years). The latter study also found reduced diurnal amplitude and smaller asleep-awake temperature differences in people with severe depressive symptoms. Differences between our study and others might be due to several factors. First, depression has many forms, and studies like this one designed to examine people early in the course of illness and also looks at separate types based on physiological differences are not common. Beyond this, skin temperature is highly sensitive to environmental conditions, such as ambient temperature and activity levels, which were uncontrolled in our study. This can be exacerbated by contact inconsistency from wrist-form sensors (Smarr et al., 2016). In addition, while the GENEActiv wearable device has a manufacturer-validated temperature sensor, it has not been validated against gold-standard temperature measurement. As reported in the Supplementary Materials (Figures S1-S3), our within-person comparison between GENEActiv and iButton sensors showed similar rhythmic patterns but different absolute temperature values. Another possible source of discrepancy is that we focused on cosinor-based rhythmic patterns rather than time-specific temperature variation. Finally, differences in age (and hence in physiological profiles across study populations) could further explain these divergent findings. Nevertheless, all these studies support the view that disruptions to circadian rhythms and temperature regulation (which may be causally related) provide valuable insights into people with depressive disorders.

Our analysis of skin temperature rhythmicity parameters provides additional insights into circadian disruptions in youth with emerging mood disorders. The mood disorder cohort had delayed acrophase (the timing of peak skin temperature), reduced inter-daily stability (IS), and increased intra-daily variability (IV), indicating more fragmented and less consistent 24-h temperature rhythms. Regarding sleep-wake parameters, we observed lower SRI values and lower moderate-to-vigorous physical activity (MVPA) in the mood disorder group in the absence of a significant delay in sleep midpoint. This lower IS may reflect weaker synchronization between the circadian system and external cues, such as the light-dark cycle, which could exacerbate problems with mood and other symptoms. Higher IV, indicating greater fragmentation of the temperature rhythm, could suggest autonomic dysregulation (Kalsbeek et al., 2011; Moraes et al., 2013). These analyses also revealed a larger phase angle difference in the mood disorder group, indicating that their peak skin temperature occurred later relative to their sleep midpoint compared to healthy controls. This misalignment between temperature rhythms and sleep timing could be a key factor in the sleep disturbances often reported in mood disorders (e.g., unrefreshing sleep, insomnia/hypersomnia).

While the patterns of skin temperature were similar across illness subtypes and clinical stages, the most notable finding was the lower relative amplitude in wrist skin temperature rhythms of the “circadian-bipolar spectrum” group, consistent with evidence of more pronounced circadian dysfunction in bipolar disorder (McCarthy et al., 2022; Scott et al., 2022). The blunted circadian rhythm in this group may indicate a weakened endogenous circadian pacemaker, which aligns with studies showing diminished circadian amplitude in mood disorders (Bullock and Murray, 2014; Castro et al., 2015; Grierson et al., 2016). Interestingly, while we observed differences in skin temperature relative amplitude between illness subtypes, most sleep-wake parameters remained similar across groups. This dissociation between temperature rhythms and sleep-wake patterns suggests a potential disruption in the normal coupling between these circadian processes. The nature of such internal misalignment is unknown, but could relate to uncoupling of neuronal oscillators within the suprachiasmatic nucleus (central pacemaker), which typically synchronizes circadian processes including sleep-wake cycles and body temperature regulation (Hut and Beersma, 2011). Such uncoupling may result in the desynchronization of circadian physiological markers and behavioral patterns in mood disorders (McCarthy and Welsh, 2012). Moreover, the findings of this study emphasize the importance of examining multiple objective signals, such as skin temperature, rather than relying solely on sleep-wake activity. Skin temperature, as a marker of autonomic and circadian regulation, provides additional insight into physiological rhythms that may not be captured by activity measures alone. Future research should explore these complex interactions, particularly the potential dissociation between central and peripheral circadian processes in cohorts with major mood disorders.

Finally, it is worth noting that in contrast to previous research (Mason et al., 2024), we did not observe relationships between symptom severity measures (e.g., BPRS, YMRS, HDRS) and skin temperature parameters in this mood disorder group (Figure S4). Future studies with larger sample sizes, and studies of different subgroups, may uncover more complex relationships between temperature parameters and clinical features.

Our study has several strengths, including the use of non-invasive, wearable, continuous skin temperature measurement in real-world conditions over several days, and the inclusion of different mood disorder subtypes also provide a more nuanced understanding of circadian disturbances in emerging mood disorders. However, the cross-sectional nature of our study limits causal conclusions about the relationship between temperature rhythms and emerging mood disorders. In addition, we did not account for the potential effects of medications, which could influence temperature regulation (Lorenz et al., 2019). While we conducted supplementary analyses controlling for BMI, the substantial difference in missing BMI data between healthy controls (63%) and mood disorder cases (25%) may limit our ability to fully understand the relationship between BMI and temperature rhythms across groups. As this was an observational study in naturalistic (real world) settings, we also did not systematically track exercise and meal timing, although these factors likely have minimal impact on skin temperature compared to core body temperature measurements. Furthermore, we did not collect information about participants’ shift work status, which could potentially influence circadian temperature rhythms.

In conclusion, our findings highlight the potential of skin temperature rhythms as a non-invasive biomarker for circadian disturbances in emerging mood disorders. The observed alterations in temperature patterns and rhythmicity offer valuable insights into the pathophysiology of these conditions, indicating that circadian misalignment may contribute to behavioral, mood, sleep, or metabolic disturbances in this population. This study emphasizes the necessity for further research to explore the implications of these findings for developing targeted interventions aimed at synchronizing circadian rhythms, ultimately enhancing treatment outcomes for young people with mood disorders.

## Supplemental Material

sj-docx-1-jbr-10.1177_07487304251328501 – Supplemental material for Twenty-four-hour Skin Temperature Rhythms in Young People With Emerging Mood Disorders: Relationships With Illness Subtypes and Clinical Stage Supplemental material, sj-docx-1-jbr-10.1177_07487304251328501 for Twenty-four-hour Skin Temperature Rhythms in Young People With Emerging Mood Disorders: Relationships With Illness Subtypes and Clinical Stage by Mirim Shin, Joanne S. Carpenter, Shin H. Park, Connie Janiszewski, Emiliana Tonini, Sarah McKenna, Gabrielle Hindmarsh, Frank Iorfino, Alissa Nichles, Natalia Zmicerevska, Elizabeth M. Scott, Benjamin L. Smarr, Ian B. Hickie and Jacob J. Crouse in Journal of Biological Rhythms

sj-txt-2-jbr-10.1177_07487304251328501 – Supplemental material for Twenty-four-hour Skin Temperature Rhythms in Young People With Emerging Mood Disorders: Relationships With Illness Subtypes and Clinical Stage Supplemental material, sj-txt-2-jbr-10.1177_07487304251328501 for Twenty-four-hour Skin Temperature Rhythms in Young People With Emerging Mood Disorders: Relationships With Illness Subtypes and Clinical Stage by Mirim Shin, Joanne S. Carpenter, Shin H. Park, Connie Janiszewski, Emiliana Tonini, Sarah McKenna, Gabrielle Hindmarsh, Frank Iorfino, Alissa Nichles, Natalia Zmicerevska, Elizabeth M. Scott, Benjamin L. Smarr, Ian B. Hickie and Jacob J. Crouse in Journal of Biological Rhythms
