# Supplementary material for: Twenty-four-hour Skin Temperature Rhythms in Young People With Emerging Mood Disorders: Relationships With Illness Subtypes and Clinical Stage
Source: J Biol Rhythms. 2025 Apr 26;40(3):262–74. doi: 10.1177/07487304251328501 (PMC12085744; doi:10.1177/07487304251328501)
Supplement: sj-docx-1-jbr-10.1177_07487304251328501 – Supplemental material for Twenty-four-hour Skin Temperature Rhythms in Young People With Emerging Mood Disorders: Relationships With Illness Subtypes and Clinical Stage [file sj-docx-1-jbr-10.1177_07487304251328501.docx]

**Comparison of GeneActiv** **and iButton temperature measurements**

Thirty-seven participants attended the Chronobiology and Sleep Laboratory at the Brain and Mind Centre within one month of the actigraphy assessment. On the day of the circadian evaluation, consumption of food or beverages containing caffeine was prohibited from noon onward. The assessment commenced approximately eight hours before participants’ habitual sleep time (HST, the average sleep onset time during the actigraphy recording period), with participants remaining seated in dim light (<30 lux) until about two hours after HST, when they were allowed to sleep until their habitual wake time (HWT, the average sleep offset time across the actigraphy recording period). Participants wore GeneActiv and iButtons on several body regions, with comparisons made between the iButton measurements on the inner forearm (closest to the wrist) and GeneActiv data. Figures S1-S3 illustrate the comparison between the skin temperature measurements from the GeneActiv and iButton devices.

**Figure S1. Bland-Altman analysis of agreement between GeneActiv and iButton skin temperature measurements**
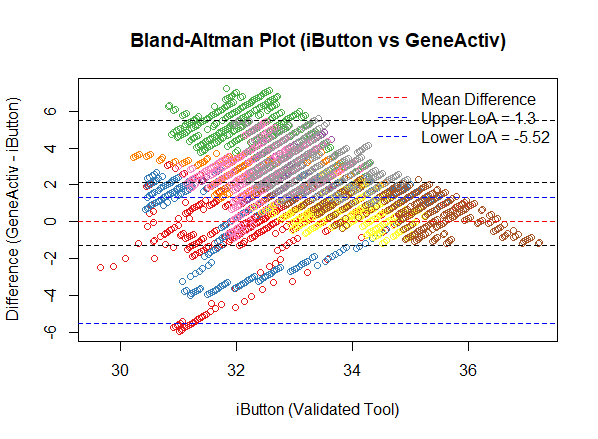


The Bland-Altman plot demonstrates the agreement between GeneActiv and iButton temperature measurements (points coloured by participant). The mean difference (bias) between devices was close to 0°C (red dashed line), indicating no systematic offset. However, the limits of agreement (LoA) ranged from -5.52°C to 1.3°C (blue dashed lines), suggesting considerable variability in agreement between devices. The plot reveals a systematic pattern where differences between devices increase with higher temperatures, indicating proportional bias. At lower temperatures (~30-32°C), GeneActiv tends to record higher values than iButton, while at higher temperatures (~34-36°C), iButton generally shows higher readings than GeneActiv. This suggests that measurement agreement is temperature-dependent.

**Figure S2. Individual skin temperature profiles comparing simultaneous GeneActiv and iButton recordings**


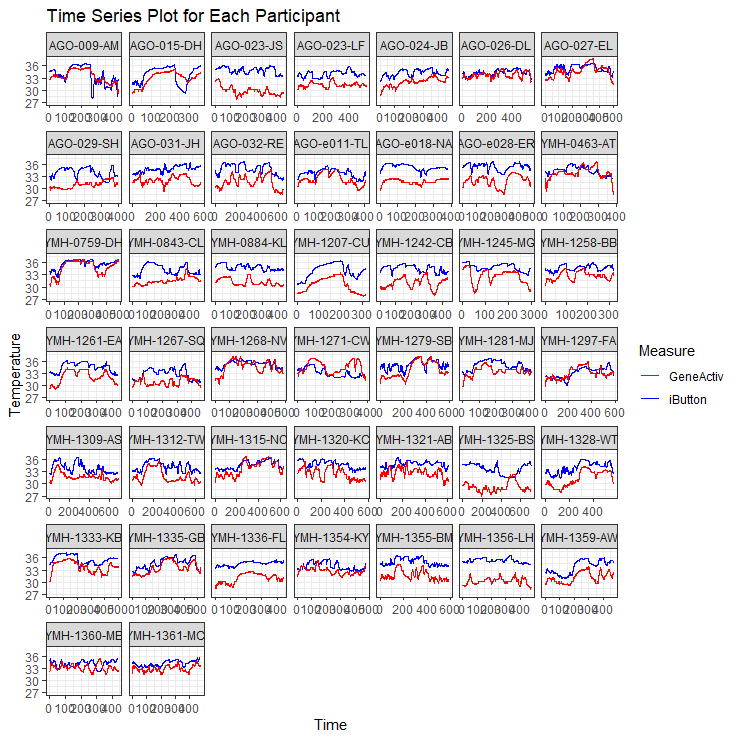


Time series plots for each participant (n=37) show concurrent temperature measurements from GeneActiv (red) and iButton (blue) devices. While both devices captured similar temporal patterns and rhythmicity, there were individual variations in the magnitude of agreement. Some participants showed good agreement between devices (e.g., YMH-1315-NO), while others demonstrated more substantial differences (e.g., YMH-1325-BS).

**Figure S3. Aggregated mean skin temperature profiles from GeneActiv and iButton recordings**


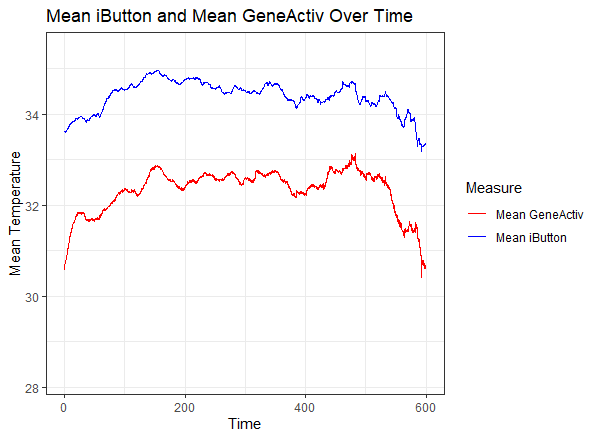


The averaged temperature profiles demonstrate overall temporal patterns captured by both devices across the recording period. iButton measurements (blue line) consistently showed higher absolute temperature values than GeneActiv (red line), with an average difference of approximately 2°C. Despite this offset, both devices captured similar temporal dynamics and patterns of temperature fluctuation. The parallel nature of the curves suggests that while absolute values differ, relative changes in temperature are consistently detected by both devices.

**Figure S4. Correlation matrix of clinical measures, sleep parameters, and circadian variables in people with mood disorders**
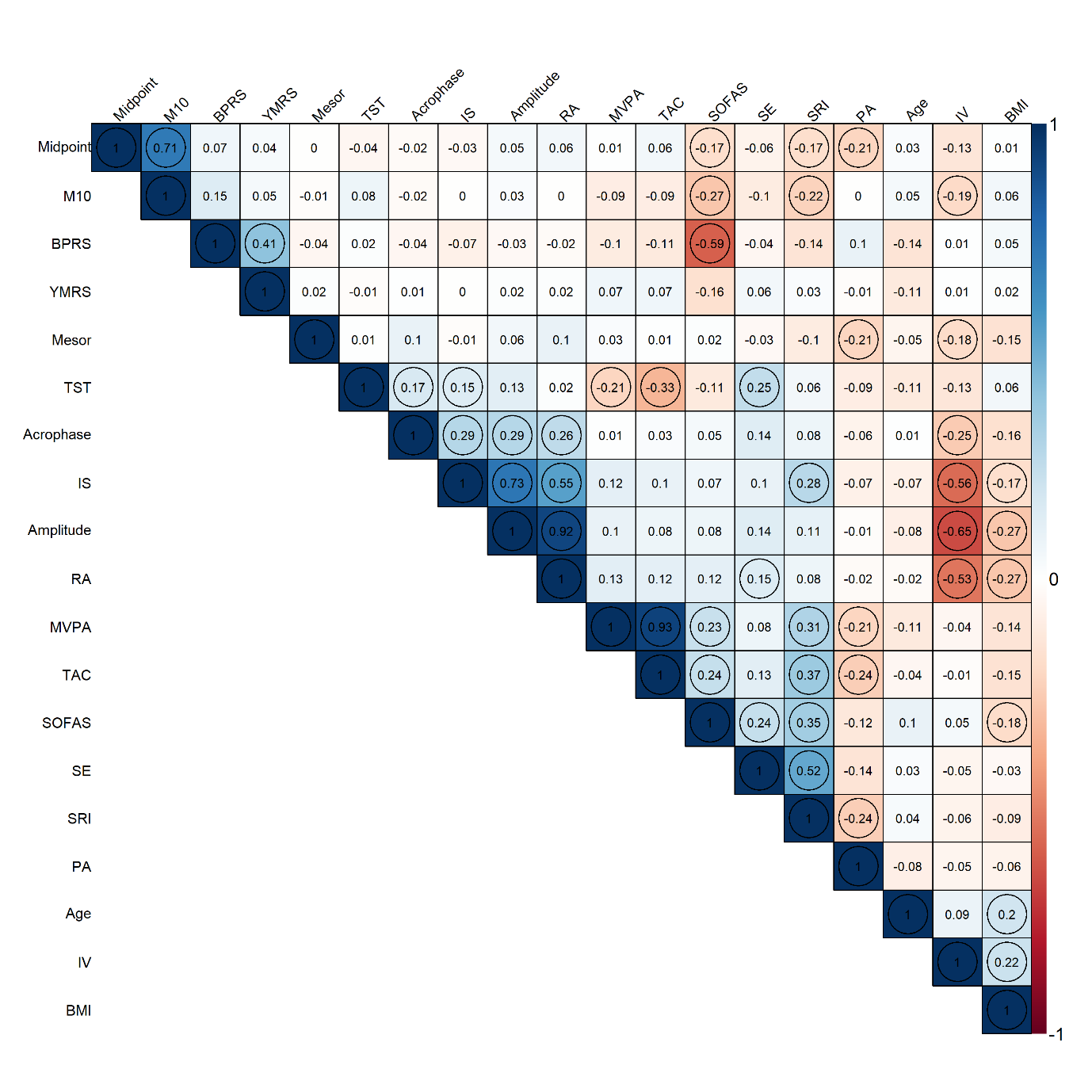


Correlation analyses revealed significant relationships among temperature circadian parameters: skin temperature interdaily stability (IS) showed strong positive correlations with amplitude (r=0.73) and relative amplitude (RA) (r=0.55), while showing negative correlation with intradaily variability (IV) (r=-0.56). Skin temperature acrophase was moderately correlated with IS (r=0.29), amplitude (r=0.29), and RA (r=0.26), and negatively correlated with IV (r=-0.25). Additionally, temperature acrophase showed a positive correlation with total sleep time (r=0.17) and negative correlation with BMI (r=-0.16). Psychiatric assessments (BPRS, YMRS, and SOFAS) were not significantly correlated with any of skin temperature parameters.

**Figure S5. Correlations between number of days of skin temperature data and rhythmic parameters**

**
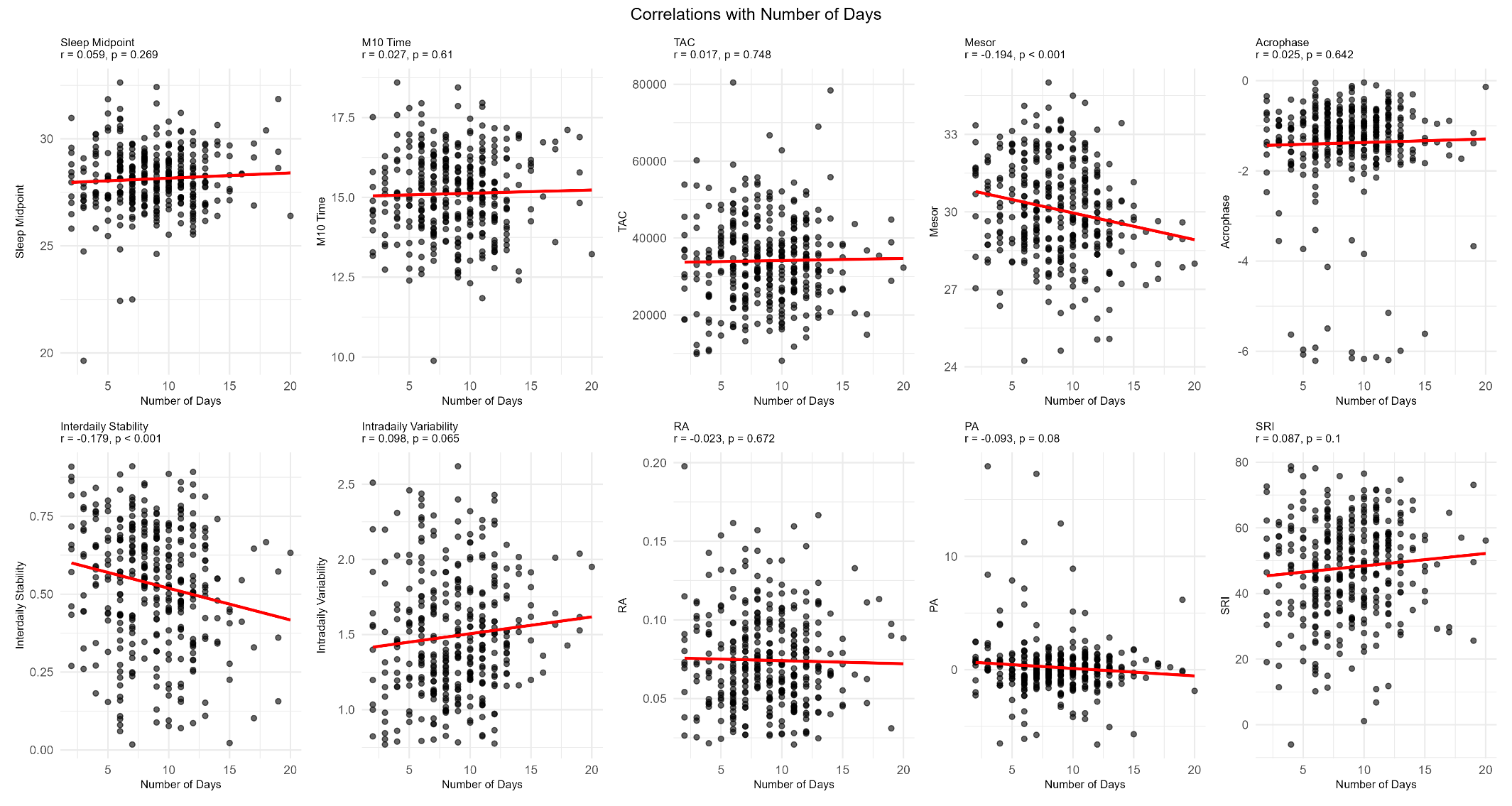
**

**Table S1. Demographic and clinical comparison across illness subtypes.**

|  | Hyperarousal Anxious-Depression  (n=209) | Circadian-Bipolar Spectrum  (n=43) | Neurodevelopmental-Psychosis  (n=40) | Kruskal-Wallis/χ^2^ |
| --- | --- | --- | --- | --- |
| Age | 23.19 (4.99) | 23.65 (4.94) | 24.20 (4.56) | Χ^2^(2)=2.29, 0.319 |
| **Sex (Female)** | **137F (66%)** | **33F (77%)** | **20F (50%)** | Χ^2^(2)=6.60, **0.037** |
| Recording duration (days) | 8.81 (3.50) | 7.84 (3.18) | 8.50 (4.28) | Χ^2^(2)=2.95, 0.229 |
| BMI  (% missing) | 24.53 (5.16)  (25%) | 26.11 (5.06)  (28%) | 24.81 (5.13)  (10%) | Χ^2^(2)=2.77, 0.250 |
| **Clinical Stages** | **1 missing** |  |  | **Χ^2^(6)=51.32, <0.001** |
| **Stage 1a** | **42 (20%)** | **0 (0%)** | **3 (8%)** |  |
| **Stage 1b** | **134 (64%)** | **16 (37%)** | **21 (53%)** |  |
| **Stage 2+** | **32 (15%)** | **27 (63%)** | **16 (40%)** |  |
| BPRS  (% missing) | 36.01 (6.60)  (18%) | 38.09 (6.93)  (26%) | 37.61 (7.09)  (5%) | Χ^2^(2)=3.57, 0.168 |
| **YMRS**  **(% missing)** | **1.46 (1.63)***  **(21%)** | **2.30 (3.27)**  **(30%)** | **2.67 (3.01)***  **(10%)** | **Χ^2^(2)=7.64, 0.022** |
| SOFAS  (% missing) | 63.02 (14.33)  (1%) | 63.23 (13.05)  (0%) | 60.88 (13.75)  (0%) | Χ^2^(2)=0.99, 0.609 |

Data are presented as mean (standard deviation).

**Table S2. Demographic and clinical comparison across illness stages.**

|  | Stage 1a  (n=47) | Stage 1b  (n=173) | Stage 2+  (n=76) | Kruskal-Wallis/χ^2^ |
| --- | --- | --- | --- | --- |
| Age | 22.74 (5.21) | 23.24 (4.82) | 23.96 (5.01) | Χ^2^(2)=2.64, 0.267 |
| **Sex (Female)** | 25F (53%) | 114F (66%) | 52F (68%) | Χ^2^(2)=3.28, 0.194 |
| Recording duration (days) | 8.55 (3.51) | 8.96 (3.67) | 7.96 (3.42) | Χ^2^(2)=2.64, 0.267 |
| BMI  (% missing) | 24.36 (4.88)  (19%) | 24.22 (4.87)  (20%) | 26.32 (6.13)  (34%) | Χ^2^(2)=4.80, 0.091 |
| **Illness subtypes** | **2 missing** | **1 missing** | **1 missing** | **Χ^2^(4)=49.50, <0.001** |
| **Hyperarousal**  **anxious-depression** | **42 (89%)** | **134 (77%)** | **32 (42%)** |  |
| **Circadian-bipolar**  **spectrum** | **0 (0%)** | **17 (10%)** | **27 (36%)** |  |
| **Neurodevelopmental**  **psychosis** | **3 (6%)** | **21 (12%)** | **16 (21%)** |  |
| **BPRS**  (% missing) | **32.48 (5.69)*^#^**  (15%) | **36.61 (6.22)***  (16%) | **38.90 (7.46) ^#^**  (22%) | **Χ^2^(2)=24.06, <0.001** |
| **YMRS**  (% missing) | **1.18 (1.47)***  (15%) | **1.66 (2.22)^#^**  (20%) | **2.38 (2.53)*^#^**  (28%) | **Χ^2^(2)=10.64, 0.005** |
| **SOFAS**  (% missing) | **73.17 (12.17)*^#^**  (0%) | **62.94 (12.84)*^†^**  (1%) | **56.70 (14.18)^#†^**  (0%) | **Χ^2^(2)=38.09, <0.001** |

**Table S3. Skin temperature, and sleep-wake parameters in youth with mood disorders vs healthy controls, controlling for age, sex and BMI.**

|  | Controls (n=18) | Mood disorders  (n=231) | ANCOVA |
| --- | --- | --- | --- |
| Skin temperature parameters |  |  |  |
| **Mesor** | **30.60 (0.41)** | **29.58 (0.11)** | **F(1,244)=5.80, 0.017** |
| Amplitude | 2.21 (0.23) | 2.01 (0.06) | F(1,244)=0.70, 0.402 |
| Acrophase | -1.01 (0.23) | -1.40 (0.06) | F(1,244)=2.76, 0.098 |
| IS | 0.58 (0.04) | 0.53 (0.01) | F(1,244)=1.56, 0.213 |
| IV | 1.37 (0.09) | 1.50 (0.03) | F(1,244)=1.79, 0.182 |
| RA | 0.08 (0.007) | 0.08 (0.002) | F(1,244)=0.02, 0.898 |
|  |  |  |  |
| Sleep-wake parameters |  |  |  |
| Sleep efficiency | 86.0 (1.2) | 84.6 (0.3) | F(1,244)=1.29, 0.257 |
| Sleep midpoint | 04:13 (00:20) | 04:12 (00:06) | F(1,244)=0.004, 0.948 |
| Sleep duration | 7.43 (0.27) | 7.40 (0.07) | F(1,244)=0.01, 0.913 |
| **SRI** | 55.02 (3.31) | 47.87 (0.92) | **F(1,244)=4.32, 0.039** |
| M10 time | 15:31 (00:19) | 15:08 (00:05) | F(1,244)=1.32, 0.251 |
| MVPA | 77.78 (9.23) | 77.03 (2.56) | F(1,244)=0.01, 0.938 |
| TAC | 34167.77 (2555.55) | 33809.33 (707.97) | F(1,244)=0.02, 0.893 |
|  |  |  |  |
| **Phase Angle** | **-0.74 (0.54)** | **0.44 (0.15)** | **F(1,244)=4.39, 0.037** |

Note: Data are presented as adjusted mean (Standard error); IS = Inter-daily stability; IV = Intra-daily variability; RA = Relative amplitude (M5/L10); SRI = Sleep Regularity Index; MVPA = Moderate/vigorous physical activity; TAC = Total activity count

**Table S4. Skin temperature and sleep-wake parameters across illness subtypes, controlling for age, sex and BMI.**

|  | Hyperarousal Anxious-Depression  (n=157) | | Circadian-Bipolar Spectrum  (n=31) | | Neurodevelopmental-Psychosis  (n=36) | ANCOVA |
| --- | --- | --- | --- | --- | --- | --- |
| Skin temperature parameters | |  | |  | |  |
| Mesor | 29.55 (0.14) | | 29.81 (0.31) | | 29.06 (0.29) | F(2,218)=1.78, 0.171 |
| Amplitude | 1.96 (0.08) | | 1.93 (0.17) | | 2.34 (0.16) | F(2,218)=2.52, 0.083 |
| Acrophase | -1.45 (0.08) | | -1.25 (0.18) | | -1.41 (0.17) | F(2,218)=0.50, 0.606 |
| IS | 0.51 (0.02) | | 0.54 (0.03) | | 0.56 (0.03) | F(2,218)=1.34, 0.264 |
| IV | 1.51 (0.03) | | 1.54 (0.07) | | 1.39 (0.07) | F(2,218)=1.66, 0.194 |
| **RA** | **0.07 (0.005)** | | **0.08 (0.002)** | | **0.09 (0.005)** | **F(2,218)=3.07, 0.048** |
|  |  | |  | |  |  |
| Sleep-wake parameters | |  | |  | |  |
| Sleep efficiency | 84.4 (0.4) | | 85.3 (1.0) | | 84.9 (0.9) | F(2,218)=0.48, 0.619 |
| **Sleep midpoint** | **03:58 (00:15)^*^** | | **04:07 (00:07)** | | **04:45 (00:14)^*^** | **F(2,218)=3.41, 0.035** |
| **Sleep duration** | **7.27 (0.09)** | | **7.74 (0.21)** | | **7.64 (0.19)** | **F(2,218)=3.14, 0.045** |
| SRI | 47.15 (1.13) | | 48.77 (2.57) | | 47.86 (2.38) | F(2,218)=0.18, 0.833 |
| M10 time | 15:08 (00:15) | | 15:02 (00:06) | | 15:35 (00:13) | F(2,218)=2.37, 0.096 |
| MVPA | 79.48 (3.16) | | 72.01 (7.17) | | 66.27 (6.65) | F(2,218)=1.82, 0.164 |
| TAC | 34560.62 (872.71) | | 32743.59 (1976.89) | | 30294.49 (1834.82) | F(2,218)=2.32, 0.100 |
|  |  | |  | |  |  |
| Phase Angle | 0.46 (0.19) | | 0.28 (0.43) | | 0.60 (0.40) | F(2,218)=0.15, 0.865 |

Note: Data are presented as adjusted mean (Standard error); IS = Inter-daily stability; IV = Intra-daily variability; RA = Relative amplitude (M5/L10); SRI = Sleep Regularity Index; MVPA = Moderate/vigorous physical activity; TAC = Total activity count

**Table S5. Skin temperature and sleep-wake parameters across illness stages, controlling for age, sex and BMI.**

|  | Stage 1a  (n=38) | Stage 1b  (n=139) | Stage 2+  (n=50) | ANCOVA |
| --- | --- | --- | --- | --- |
| Skin temperature parameters | |  |  |  |
| Mesor | 29.09 (0.28) | 29.65 (0.15) | 29.62 (0.25) | F(2,221)=1.60, 0.204 |
| Amplitude | 1.85 (0.16) | 1.97 (0.08) | 2.21 (0.14) | F(2,221)=1.68, 0.189 |
| Acrophase | -1.32 (0.16) | -1.42 (0.09) | -1.42 (0.14) | F(2,221)=0.20, 0.822 |
| IS | 0.49 (0.03) | 0.53 (0.02) | 0.53 (0.03) | F(2,221)=0.86, 0.427 |
| IV | 1.56 (0.06) | 1.50 (0.03) | 1.46 (0.06) | F(2,221)=0.60, 0.552 |
| RA | 0.08 (0.005) | 0.08 (0.002) | 0.08 (0.004) | F(2,221)=0.95, 0.388 |
|  |  |  |  |  |
| Sleep-wake parameters |  |  |  |  |
| Sleep efficiency | 86.0 (0.9) | 84.2 (0.4) | 84.5 (0.8) | F(2,221)=1.83, 0.163 |
| Sleep midpoint | 03:50 (00:14) | 04:16 (00:07) | 04:18 (00:12) | F(2,221)=1.48, 0.231 |
| **Sleep duration** | **6.93 (0.18)*** | **7.38 (0.10)^#^** | **7.84 (0.16)*^#^** | **F(2,221)=6.95, 0.001** |
| SRI | 50.78 (2.31) | 46.94 (1.20) | 47.26 (2.03) | F(2,221)=1.12, 0.329 |
| **M10 time** | **14:35 (00:13)^*#^** | **15:14 (00:07)^*^** | **15:20 (00:11)^#^** | **F(2,221)=4.22, 0.016** |
| **MVPA** | **98.45 (6.31)^*#^** | **72.18 (3.30)^*^** | **73.33 (5.57)^#^** | **F(2,221)=7.05, 0.001** |
| **TAC** | **40721.32 (1721.92)^*#^** | **32365.06 (898.80)^*^** | **32245.03 (1518.15)^#^** | **F(2,221)=9.85, <0.001** |
|  |  |  |  |  |
| Phase Angle | 0.45 (0.38) | 0.61 (0.20) | -0.05 (0.34) | F(2,221)=1.36, 0.260 |

Note: Data are presented as adjusted mean (Standard error); IS = Inter-daily stability; IV = Intra-daily variability; RA = Relative amplitude (M5/L10); SRI = Sleep Regularity Index; MVPA = Moderate/vigorous physical activity; TAC = Total activity count

**Table S6. Demographics, skin temperature, and sleep-wake parameters in youth with mood disorders vs healthy controls (analysis restricted to participants with ≥5 days of completed skin temperature data).**

|  | Healthy controls (n=48) | Mood disorders  (n=264) | Mann-Whitney/χ^2^ |
| --- | --- | --- | --- |
| Age (y) | 24.44 (3.38) | 23.55 (4.90) | U=5471.5, 0.132 |
| Sex (Female (%)) | 29F(60) | 172F (65) | Χ^2^(1)=0.397, 0.528 |
| Recording duration (days) | 9.21 (2.45) | 9.51 (3.02) | U=6201.5, 0.814 |
| **BMI (kg/m^2^)**  **(% missing BMI)** | **22.15 (3.62)**  **(63%)** | **24.70 (5.19)**  **(23%)** | **U=1309.0, 0.049** |
|  |  |  | ANCOVA |
| *Skin temperature parameters* |  |  |  |
| **Mesor (°C)** | **31.04 (0.25)** | **29.89 (0.11)** | **F(1,308)=17.37, <0.001** |
| Amplitude (°C) | 2.07 (0.14) | 1.94 (0.06) | F(1,308)=0.78, 0.379 |
| **Acrophase (radians)** | **-0.93 (0.16)** | **-1.46 (0.07)** | **F(1,308)=9.22, 0.003** |
| **IS (dimensionless, 0-1)** | **0.60 (0.03)** | **0.51 (0.01)** | **F(1,308)=9.57, 0.002** |
| **IV (dimensionless, 0-2)** | **1.37 (0.06)** | **1.53 (0.02)** | **F(1,308)=6.80, 0.010** |
| RA (dimensionless, 0-1) | 0.07 (0.004) | 0.07 (0.002) | F(1,308)=0.50, 0.480 |
|  |  |  |  |
| *Sleep-wake parameters* |  |  |  |
| Sleep efficiency (%) | 84.8 (0.8) | 84.2 (0.3) | F(1,308)=0.48, 0.487 |
| Sleep midpoint (time) | 04:03 (00:12) | 04:12 (00:05) | F(1,308)=0.45, 0.501 |
| Sleep duration (h) | 7.20 (0.14) | 7.27 (0.06) | F(1,308)=0.22, 0.638 |
| **SRI (0-100)** | **54.77 (2.06)** | **46.76 (0.87)** | **F(1,308)=12.85, <0.001** |
| M10 time (time) | 15:12 (00:11) | 15:07 (00:05) | F(1,308)=0.21, 0.649 |
| MVPA (minutes/day) | 86.86 (5.83) | 78.46 (2.48) | F(1,308)=1.76, 0.186 |
| **TAC (counts/day)** | **37051.64 (1550.30)** | **34034.62 (659.69)** | **F(1,308)=3.20, 0.075** |
|  |  |  |  |
| ***Phase Angle (radians)*** | **-0.49 (0.32)** | **0.18 (0.14)** | **F(1,308)=3.68, 0.056** |

**Note**: For Mann-Whitney/χ^2^, data are presented as mean (standard deviation). For ANCOVA results, data are presented as estimated mean (standard error). IS = Inter-daily stability; IV = Intra-daily variability; RA = Relative amplitude; SRI = Sleep Regularity Index; MVPA = Moderate/vigorous physical activity; TAC = Total activity count

**Table S7. Skin temperature and sleep-wake parameters across illness subtypes (analysis restricted to participants with ≥5 days of completed skin temperature data).**

|  | Hyperarousal Anxious-Depression  (n=186) | | Circadian-Bipolar Spectrum  (n=36) | | Neurodevelopmental-Psychosis  (n=30) | ANCOVA |
| --- | --- | --- | --- | --- | --- | --- |
| *Skin temperature parameters* | |  | |  | |  |
| Mesor (°C) | 29.82 (0.13) | | 30.20 (0.30) | | 29.27 (0.32) | F(2,247)=2.23, 0.109 |
| Amplitude (°C) | 1.97 (0.07) | | 1.78 (0.16) | | 2.04 (0.18) | F(2,247)=0.69, 0.501 |
| Acrophase  (radians) | -1.45 (0.09) | | -1.52 (0.20) | | -1.41 (0.22) | F(2,247)=0.07, 0.937 |
| IS (0-1) | 0.50 (0.01) | | 0.52 (0.03) | | 0.52 (0.04) | F(2,247)=0.19, 0.829 |
| IV (0-2) | 1.51 (0.03) | | 1.62 (0.07) | | 1.48 (0.07) | F(2,247)=1.24, 0.292 |
| RA (0-1) | 0.08 (0.002) | | 0.07 (0.005) | | 0.08 (0.005) | F(2,247)=1.62, 0.200 |
|  |  | |  | |  |  |
| *Sleep-wake parameters* | |  | |  | |  |
| Sleep  efficiency (%) | 84.2 (0.9) | | 84.1 (0.4) | | 84.7 (1.0) | F(2,247)=0.15, 0.859 |
| **Sleep**  **midpoint (time)** | **04:06 (00:06)*** | | **04:04 (00:14)^#^** | | **04:56 (00:16)*^#^** | F(2,247)=4.46, **0.012** |
| Sleep  duration (h) | 7.24 (0.08) | | 7.35 (0.17) | | 7.40 (0.19) | F(2,247)=0.47, 0.623 |
| SRI (0-100) | 45.85 (1.09) | | 47.48 (2.50) | | 48.75 (2.73) | F(2,247)=0.59, 0.554 |
| **M10 time**  **(time)** | **15:01 (00:06)*** | | **15:09 (00:13)** | | **15:40 (00:15)*** | **F(2,247)=3.04, 0.050** |
| MVPA  (minutes/day) | 78.83 (2.97) | | 76.92 (6.78) | | 66.37 (7.41) | F(2,247)=1.22, 0.297 |
| TAC  (counts/day) | 34052.73 (791.61) | | 34302.81  (1809.46) | | 31178.51 (1978.48) | F(2,247)=0.96, 0.384 |
|  |  | |  | |  |  |
| *Phase Angle (radians)* | 0.28 (0.18) | | -0.22 (0.40) | | 0.43 (0.44) | F(2,247)=0.76, 0.471 |

**Note**: Data are presented as estimated mean (standard error). Matching symbols indicate significant differences (p<0.05) in post-hoc pairwise comparisons between groups. IS = Inter-daily stability; IV = Intra-daily variability; RA = Relative amplitude; SRI = Sleep Regularity Index; MVPA = Moderate/vigorous physical activity; TAC = Total activity count

**Table S8. Skin temperature and sleep-wake parameters across illness stages (analysis restricted to participants with ≥5 days of completed skin temperature data).**

|  | Stage 1a  (n=41) | Stage 1b  (n=152) | Stage 2+  (n=62) | ANCOVA |
| --- | --- | --- | --- | --- |
| *Skin temperature parameters* | |  |  |  |
| **Mesor (°C)** | **29.12 (0.28)^*#^** | **29.94 (0.14)^*^** | **30.12 (0.23)^#^** | **F(2,250)=4.30, 0.015** |
| Amplitude (°C) | 1.88 (0.16) | 2.00 (0.08) | 1.85 (0.13) | F(2,250)=0.64, 0.527 |
| Acrophase (radians) | -1.41 (0.19) | -1.45 (0.10) | -1.47 (0.15) | F(2,250)=0.04, 0.965 |
| IS (0-1) | 0.49 (0.03) | 0.52 (0.02) | 0.48 (0.03) | F(2,250)=1.04, 0.357 |
| IV (0-2) | 1.56 (0.06) | 1.51 (0.03) | 1.55 (0.05) | F(2,250)=0.36, 0.701 |
| RA (0-1) | 0.08 (0.005) | 0.08 (0.002) | 0.07 (0.004) | F(2,250)=0.57, 0.569 |
|  |  |  |  |  |
| *Sleep-wake parameters* |  |  |  |  |
| **Sleep efficiency (%)** | **86.0 (0.8)^*^** | **84.1 (0.4)** | **83.3 (0.7)^*^** | **F(2,250)=3.26, 0.040** |
| Sleep midpoint (time) | 03:53 (00:14) | 04:13 (00:07) | 04:23 (00:11) | F(2,250)=1.38, 0.255 |
| **Sleep duration (h)** | **6.84 (0.16)^*#^** | **7.31 (0.08)^*^** | **7.47 (0.13)^#^** | **F(2,250)=5.02, 0.007** |
| **SRI (0-100)** | **52.77 (2.30)^*#^** | **45.93 (1.18)^*^** | **43.89 (1.86)^#^** | **F(2,250)=4.75, 0.009** |
| **M10 time (time)** | **14:30 (00:12)^*#^** | **15:08 (00:06)^*^** | **15:26 (00:10)^#^** | **F(2,250)=6.05, 0.003** |
| **MVPA**  **(minutes/day)** | **98.39 (6.54)^*#^** | **74.67 (3.36)^*^** | **73.85 (5.29)^#^** | **F(2,250)=5.61, <0.001** |
| **TAC (counts/day)** | **40227.44 (1701.36)^*#^** | **32871.34 (874.76)^*^** | **32623.65 (1377.05)^#^** | **F(2,291)=7.97, <0.001** |
|  |  |  |  |  |
| *Phase Angle (radians)* | 0.24 (0.37) | 0.40 (0.19) | -0.26 (0.30) | F(2,250)=1.67, 0.191 |

**Note**: Data are presented as estimated mean (standard error). Matching symbols indicate significant differences (p<0.05) between groups in post-hoc pairwise comparisons. IS = Inter-daily stability; IV = Intra-daily variability; RA = Relative amplitude (M5/L10); SRI = Sleep Regularity Index; MVPA = Moderate/vigorous physical activity; TAC = Total activity count
